# Chloroplast genome characteristics and phylogenetic analysis of *Mangifera indica* L. ‘Guiqi’ and *M. quadrifida* Jack (anacardiaceae)

**DOI:** 10.1080/23802359.2025.2550612

**Published:** 2025-09-29

**Authors:** Kaikai Meng, Yuming Qin, Junru Qu, Shiou Yih Lee, Liyan Lu, Peishan Zou, Guodi Huang

**Affiliations:** ^a^ Guangxi Key Laboratory of Quality and Safety Control for Subtropical Fruits/Key Laboratory of Quality and Safety Control for Subtropical Fruit and Vegetable, Ministry of Agriculture and Rural Affairs, Guangxi Subtropical Crops Research Institute, Nanning, Guangxi, China; ^b^ Guangxi Zhuang Autonomous Region Engineering Research Center of Green and Efficient Development for Mango Industry, Guangxi Subtropical Crops Research Institute, Nanning, Guangxi, China; ^c^ Faculty of Health and Life Sciences, INTI International University, Nilai, Negeri Sembilan, Malaysia; ^d^ Faculty of Nursing, Shinawatra University, Sam Khok, Pathum Thani, Thailand

**Keywords:** Mangifera, plastid genome, genetic resources, phylogeny

## Abstract

Despite being an economically significant fruit, the availability of *Mangifera* chloroplast genome sequences remains limited. In this study, we assembled, annotated, and characterized the complete chloroplast genomes of *Mangifera indica* ‘Guiqi’ and *M. quadrifida*. The chloroplast genomes of *M. indica* ‘Guiqi’ (157,780 bp) and *M. quadrifida* (158,979 bp) both exhibit a quadripartite structure, contain 87 protein-coding, 37 tRNA, and 8 rRNA genes, and have GC contents of 37.9% and 37.8%, respectively. Phylogenetic analysis confirmed the maternal origin of *M. indica* ‘Guiqi’ and revealed the closest affinity between *M. quadrifida* and *M. similis*, indicating widespread hybridization and introgressions.

## Introduction

*Mangifera* L. of Anacardiaceae comprises about 68 species that are widely distributed across tropical Asia (Chase et al. 2016). They are primarily found in Malaysia, extending west to India and Sri Lanka, east to the Philippines, north through India to China, and south to Indonesia (Zheng and Min 1980). The mango (*Mangifera indica* L. 1753) is a renowned and widely popular tropical fruit, celebrated for its high nutritional value, delightful taste, and unique aroma (Liu et al. 2024), while *M. quadrifida* Jack 1824 is a rare species that grows well in areas with extreme conditions such as nutrient-poor soil and poor drainage (Rosalina et al. 2023). Both plants are highly medicinal, containing antioxidants, anti-inflammatory agents, anti-diabetic properties, and active compounds with potential anti-cancer properties (Irawan et al. 2021; Nagendla et al. 2023).

At present, a diverse range of mango cultivars are extensively cultivated across tropical and subtropical regions. A collection of genomic information on such valuable fruit crop will benefit the studies on molecular breeding, genetic conservation, and evolution (Sim et al. 2025). Despite being an important fruit crop, there are only 26 records of *Mangifera* chloroplast genomes that are currently publicly available in the NCBI GenBank database (as of November 15, 2024). This study investigated two distinct accessions: the commercial cultivar *M. indica* ‘Guiqi’ and a wild accession of *M. quadrifida*. The *M. indica* ‘Guiqi’ displayed intense aromatic attributes accompanied by exceptionally low mesocarp fiber content, whereas *M. quadrifida* manifested distinctive prune-like volatile profiles together with fibrous, edible fruits. Our objectives were to (1) assemble and annotate their complete chloroplast genomes, (2) characterize their structural features, and (3) reconstruct the phylogeny of *Mangifera* using chloroplast genome sequences.

## Materials and methods

**Figure 1.**
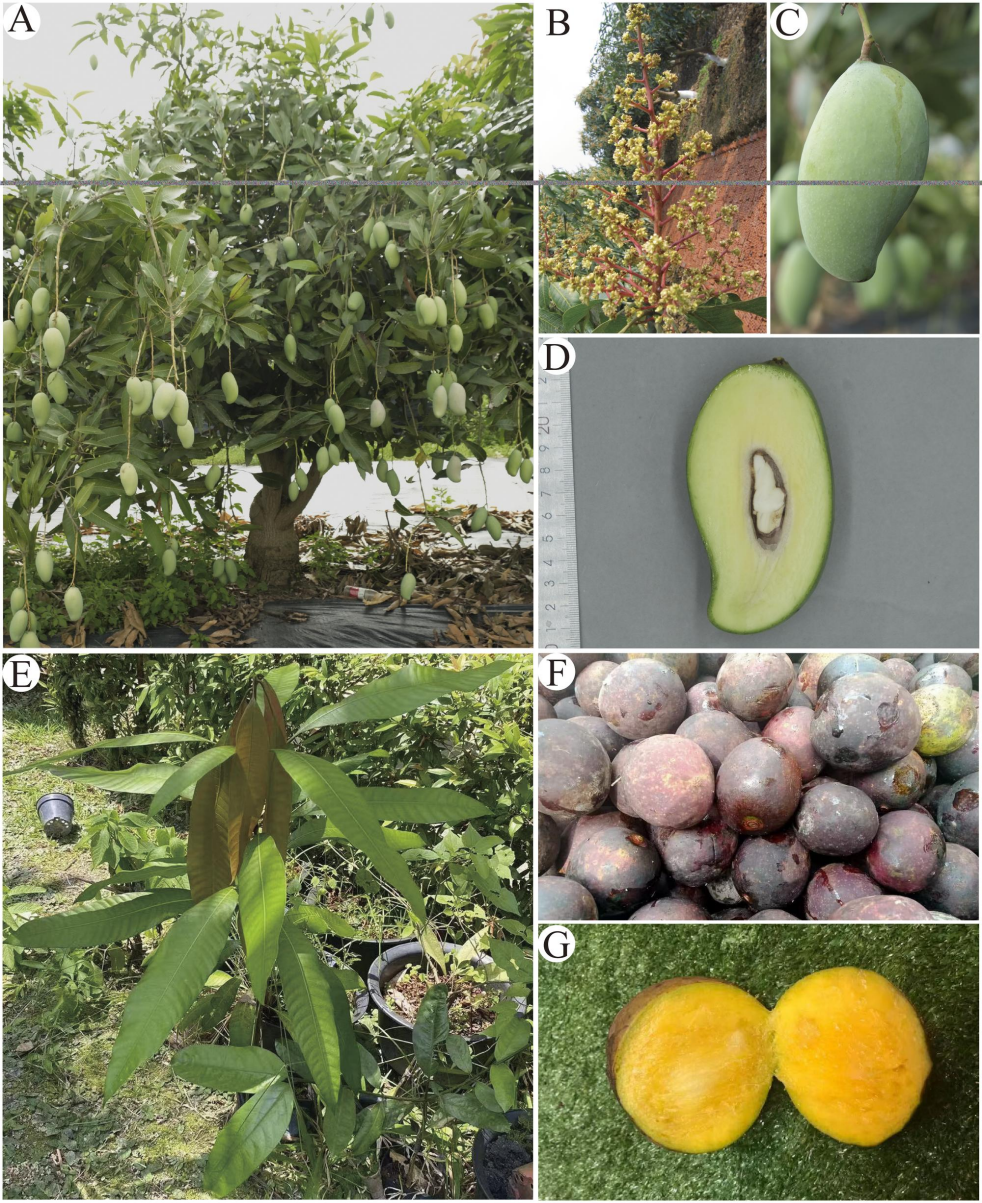
Morphological features of *Mangifera indica* ‘Guiqi’ (A–D), exhibiting intensely aromatic properties and remarkably low-fiber flesh, and *M. quadrifida* (E–G), displaying distinctive prune-like volatile compounds and characteristically fibrous white edible flesh. (A) Adult plant; (B) Inflorescence; (C) Fruit; (D) Transverse section of fruit; (E) Seedling; (F) Fruits; (G) Transverse section of fruit. Photos by G. D. Huang and S. Y. Lee.

Fresh and mature leaves were collected from an adult *M. indica* ‘Guiqi’ plant in the Germplasm Resource Nursery of Guangxi Subtropical Crops Research Institute (N22°53′55″, E108°20′33″) and from an ex-situ *M. quadrifida* accession in the TCM Herb Garden of INTI International University (2°48′41″N, 101°45′29″E). The leaves were immediately preserved in silica gel (Figure 1). The voucher specimens of *M. indica* ‘Guiqi’ and *M. quadrifia* were deposited in the herbarium at Sun Yat-sen University (http://lifesciences.sysu.edu.cn/, Prof. Dr. Wenbo Liao, lsslwb@mail.sysu.edu.cn) under the voucher number K.K. Meng 20240902 and the Biotechnology Lab of the Faculty of Health and Life Sciences, INTI International University (http://www.newinti.edu.my, Prof. Dr. Lee Shiou Yih, shiouyih.lee@newinti.edu.my) under the collection number LSY16, respectively.

The genomic DNA was obtained following the method by Meng et al. (2021). The DNA quality and quantity were assessed using a NanoDrop spectrophotometer (Thermo Fisher Scientific, USA) and Qubit fluorometer (Invitrogen, USA), respectively. DNA libraries were subsequently constructed, and paired-end (2 × 150 bp) high-throughput sequencing was performed using the DNBSEQ-T7 platform at the BGI Genomics Co., Ltd. (Guangdong, China). Raw data were processed by fastp v. 0.23.1 to generate clean data by setting -q 20 -u 10 (Chen et al. 2018), and quality control was conducted using FastQC v0.11.9 (Andrews 2010). The chloroplast genome was assembled using GetOrganelle v1.7.7.0 (Jin et al. 2020) with *rbcL* of *M. indica* (ON805861.1) as the initial seed sequence, supplemented with the embplant_pt reference database. To ensure the completeness and accuracy of the gene annotation, we manually compared the results from both tools from GeSeq v2.03 (Tillich et al. 2017) and CPGAVAS2 (Shi et al. 2019) for each genomic feature. The online software CPGView (Liu et al. 2023) was used to visualize the detailed structure of the transcripts and to identify the genes that are difficult to be annotated. Processed paired-end reads were aligned to the reference chloroplast genome using BWA-MEM v0.7.17 (Li and Durbin 2010) with default parameters. The SAM files were convert to BAM format, and then PCR duplicates were removed using SAMtools v1.10 (Li et al. 2009). The bamCoverage module in deeptools v. 3.5.1 (Ramírez et al. 2016) converted BAM files to BigWig files for visualization in Integrative Genomics Viewer (IGV) v2.11.2 (Robinson et al. 2011).

Phylogenetic analysis was conducted based on the complete chloroplast genome sequences of 26 *Mangifera* spp. available in the genbank. The closely related species, *Bouea macrophylla*, was included as outgroup. The chloroplast genome sequences were aligned using MAFFT v7 (Rozewicki et al. 2019) with the –auto parameter, and then trimmed using TrimAl v1.4 by applying the ‘gappyout’ model according to the default threshold (Capella-Gutiérrez et al. 2009). The best-fit nucleotide substitution model was calculated by ModelFinder (Kalyaanamoorthy et al. 2017). The best nucleotide substitution model was the general time reversible model (GTR) with empirical based frequencies (+F) and invariable sites (+I) (=GTR+F + I) according to the Bayesian Information Criterion (BIC). The maximum-likelihood (ML) tree was constructed using IQ-TREE v2.1 (Minh et al. 2020) by setting 10,000 replicates of Shimodaira-Hasegawa-like approximate likelihood ratio test (SH-aLRT) and 5,000 replicates of UltraFast bootstraps (UFboot) (Hoang et al. 2018).

## Results

**Figure 2.**
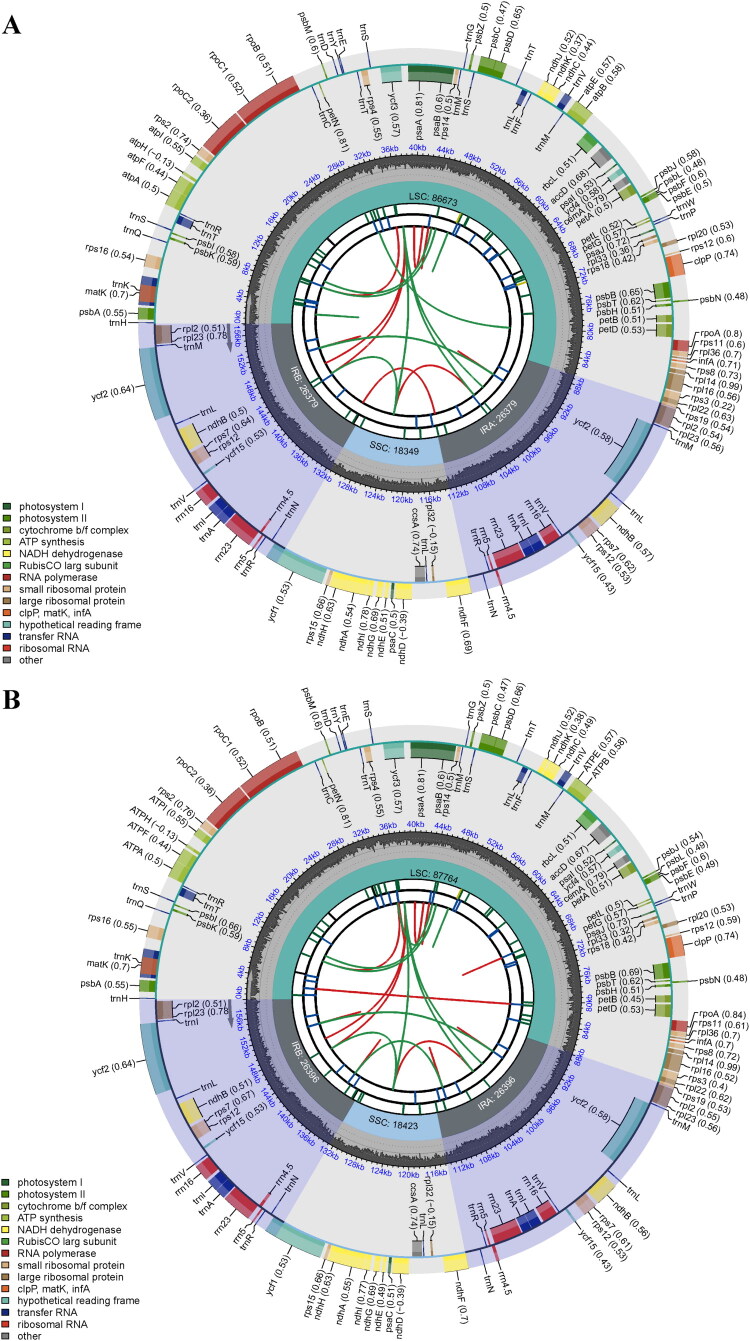
The circular maps of (A) *Mangifera indica* ‘Guiqi’ and (B) *M. quadrifida* with six circular tracks. Tracks from the center going outward represented the distributions of repeat sequences, tandem repeats, microsatellite repeats, the typical quadripartite structure, GC content, and gene distribution. The genes positioned on the outer layer are transcribed in a clockwise direction, while those on the inner layers are transcribed in a counterclockwise direction.

Sequencing generated 100,083,838 raw reads (15.01 Gb) and 98,687,824 high-quality clean reads (14.80 Gb) for *M. indica* ‘Guiqi’. For *M. quadrifia*, we obtained 86,695,480 raw reads (13.00 Gb) and 85,699,904 clean reads (12.85 Gb). Based on the reference genome of *M. indica* ‘Alphonso’ (Wang et al. 2020), the average sequencing coverage depths for the two accessions reached approximately 38×. With a mapping depth of 7,916 to 16,825, and 3,073 to 6,737 for *M. indica* ‘Guiqi’ and *M. quadrifia*, respectively (Figure S1), the assembled chloroplast genome sequences revealed quadripartite structure. The total genome sequence length, gene number, and GC content were 157,780 bp and 158,979 bp, 132 and 132, 37.9% and 37.8%, for *M. indica* ‘Guiqi’ and *M. quadrifida*, respectively. The chloroplast genomes were comprised of a large single-copy (LSC, 86,673 bp and 87,764 bp), two inverted repeat regions (IRs, 26,379 bp and 26,396 bp), and a small single-copy (SSC, 18,349 bp and 18,423 bp) (Figure 2). The chloroplast genomes of both *M. indica* ‘Guiqi’ and *M. quadrifida* contained 87 protein-coding, 37 tRNA, and 8 rRNA genes. Seventeen genes were duplicated in both species, consisting of the four rRNA genes, seven tRNA genes (*trnA-UGC*, *trnI-CAU*, *trnI-GAU*, *trnL-CAA*, *trnN-GUU*, *trnR-ACG*, and *trnV-GAC*), and six protein-coding genes (*ndhB*, *rpl2*, *rpl23*, *rps7*, *ycf2*, and *ycf15*). Nine genes, including *atpF*, *ndhA*, *ndhB*, *petB*, *petD*, *rpl2*, *rpl16*, *rpoC1*, and *rps16*, contained a single intron, whereas *clpP*, *ycf3*, and the trans-splicing gene of *rps12* possessed two introns (Figure S2, Figure S3).

**Figure 3.**
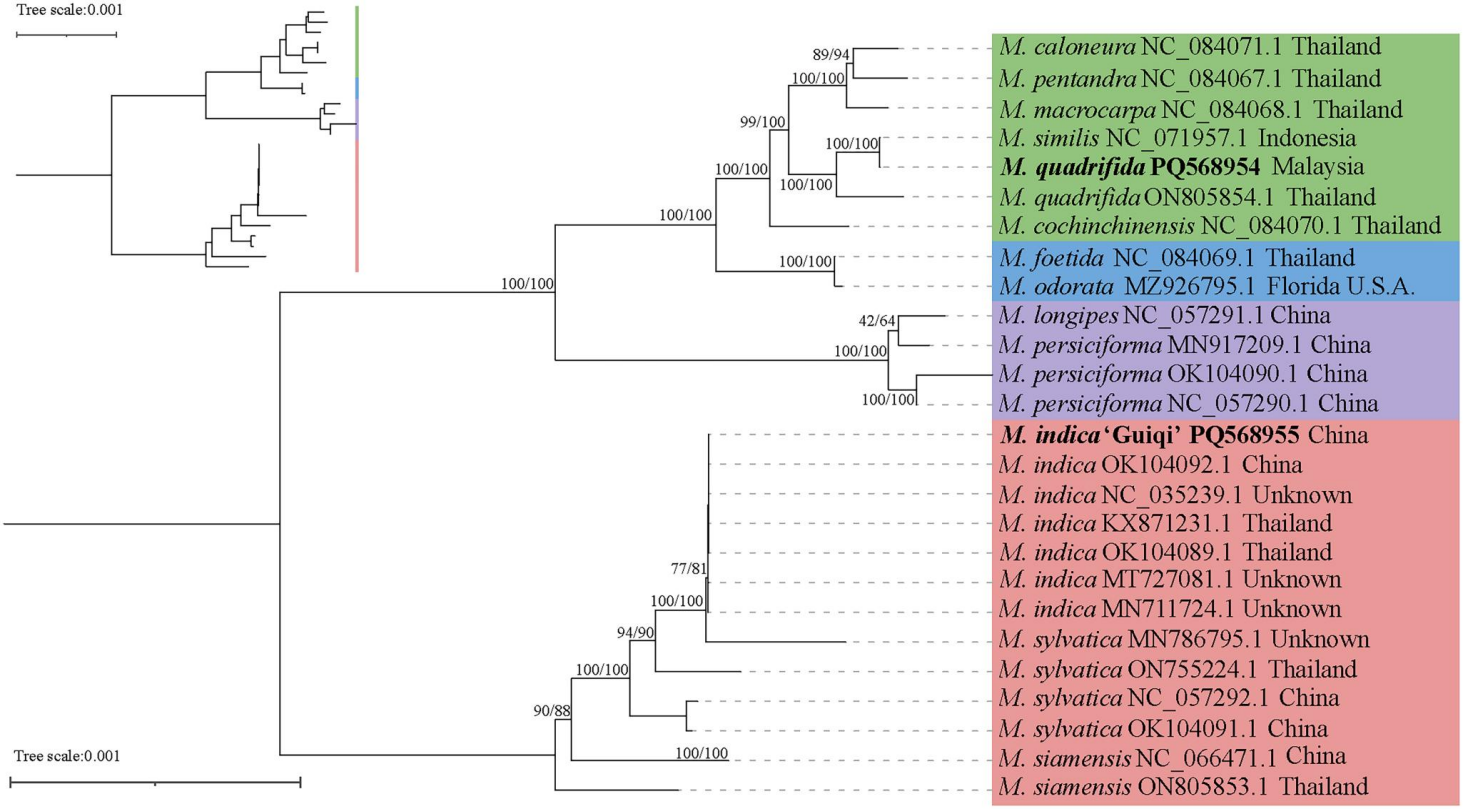
Maximum-likelihood phylogenetic tree of 26 *Mangifera* accessions and *Bouea macrophylla* as the outgroup based on the complete chloroplast genome sequences. Branch supports were calculated using the Shimodaira-Hasegawa-like approximate likelihood ratio test (SH-aLRT, left) and the UltraFast bootstraps (UFboot, right). The GenBank accession numbers and country of origin were listed behind the species name, while other information is available in table S1. The following sequences were used: *M. caloneura* (GenBank accession number: NC084071; Xin et al. 2023), *M. pentandra* (GenBank accession number: NC084067; Xin et al. 2023), *M. macrocarpa* (GenBank accession number: NC084068; Xin et al. 2023), *M. similis* (GenBank accession number: NC071957; Yuskianti et al. 2024), *M. quadrifida* (GenBank accession numbers: ON805854, PQ568954; Xin et al. 2023, this study), *M. cochinchinensis* (GenBank accession number: NC084070; Xin et al. 2023), *M. foetida* (GenBank accession number: NC084069; Xin et al. 2023), *M. odorata* (GenBank accession number: MZ926795; Tang et al. 2025), *M. longipes* (GenBank accession number: NC057291; Niu et al. 2021), *M. persiciforma* (GenBank accession numbers: MN917209, NC057290, OK104090; Niu et al. 2021, Tang et al. 2025), *M. indica* ‘Guiqi’ (GenBank accession number: PQ568955; this study), *M. indica* (GenBank accession numbers: KX871231, MN711724, MT727081, NC035239, OK104089, OK104092; Tang et al. 2025), *M. sylvatica* (GenBank accession numbers: MN786795, NC057292, OK104091; Niu et al. 2021, Tang et al. 2025), *M. siamensis* (GenBank accession numbers: NC066471, ON805853; Xin et al. 2023, Yuskianti et al. 2024).

The final trimmed alignment had a length of 157,149 bp, with 778 parsimony-informative sites identified. Phylogenetic analysis based on the complete chloroplast genome sequences revealed a partially-resolved relationship at the species level. However, the relationship was not supported at the intraspecific level, i.e. *M. indica*. The *M. indica* ‘Guiqi’ was grouped with the other six *M. indica* accessions (SH-aLRT = 77, UFboot = 81). The phylogenetic relationship of *M. quadrifida* used in this study is fully resolved as a member under the same clade as the *M. quadrifida*, and is closely related to *M. similis* (Figure 3, Figure S4).

## Discussion and conclusions

The chloroplast genomes for *M. indica* ‘Guiqi’ and *M. quadrifida* from Malaysia were newly reported, annotated, and characterized. The two chloroplast genomes were highly conserved in genome structure, GC content, gene composition, when compared to other *Mangifera* species (Xin et al. 2023; Yuskianti et al. 2024). For the *M. indica* ‘Guiqi’, the variation in the number of protein-coding genes can be attributed to the absence of the *ycf15* duplicate and *infA* genes. Based on the ML tree, the phylogenetic clustering of certain *Mangifera* species correlated more strongly with geographic distribution than taxonomic classification. Given the high heterozygosity and frequent cross-pollination observed in *Mangifera* (Wang et al. Wang et al. 2020; Ramírez and Lee 2016), such event might be attributed to the widespread introgression and hybridization occurring within or between species. Nevertheless, the chloroplast genomes assembled here will serve as a valuable resource for future mango breeding programs and germplasm development. These findings highlighted the need for future population-level studies to clarify hybridization dynamics and evolutionary mechanisms in this economically important genus.

## Supplementary Material

Supplementary materials.doc

## Data Availability

The genome sequence data that support the findings of this study are openly available in GenBank of NCBI at http://www.ncbi.nlm.nih.gov under the accession numbers PQ568955 and PQ568954. The associated BioProject, SRA, and BioSample numbers are PRJNA1176453, SRR31094034 and SRR31094035, as well as SAMN44399365 and SAMN44399366, respectively.
