# Supplementary material for: Chloroplast genome characteristics and phylogenetic analysis of Mangifera indica L. ‘Guiqi’ and M. quadrifida Jack (anacardiaceae)
Source: Mitochondrial DNA B Resour. 2025 Sep 29;10(10):996–1001. doi: 10.1080/23802359.2025.2550612 (PMC12486444; doi:10.1080/23802359.2025.2550612)
Supplement: Supplementary materials.doc [file TMDN_A_2550612_SM6959.doc]

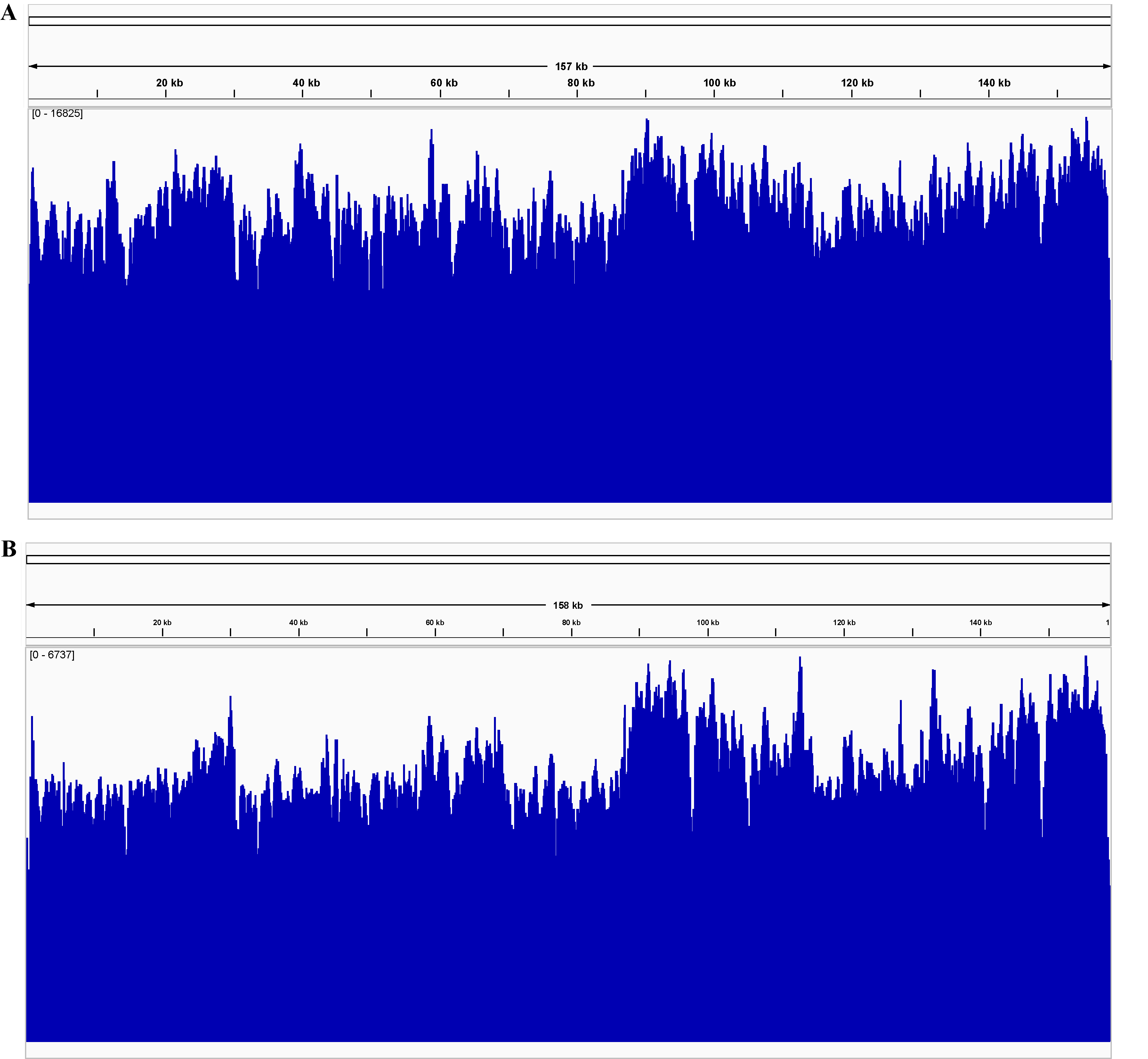


Figure S1. Sequencing coverage depth of (A) *M. indica* ‘Guiqi’ and (B) *M. quadrifida*.


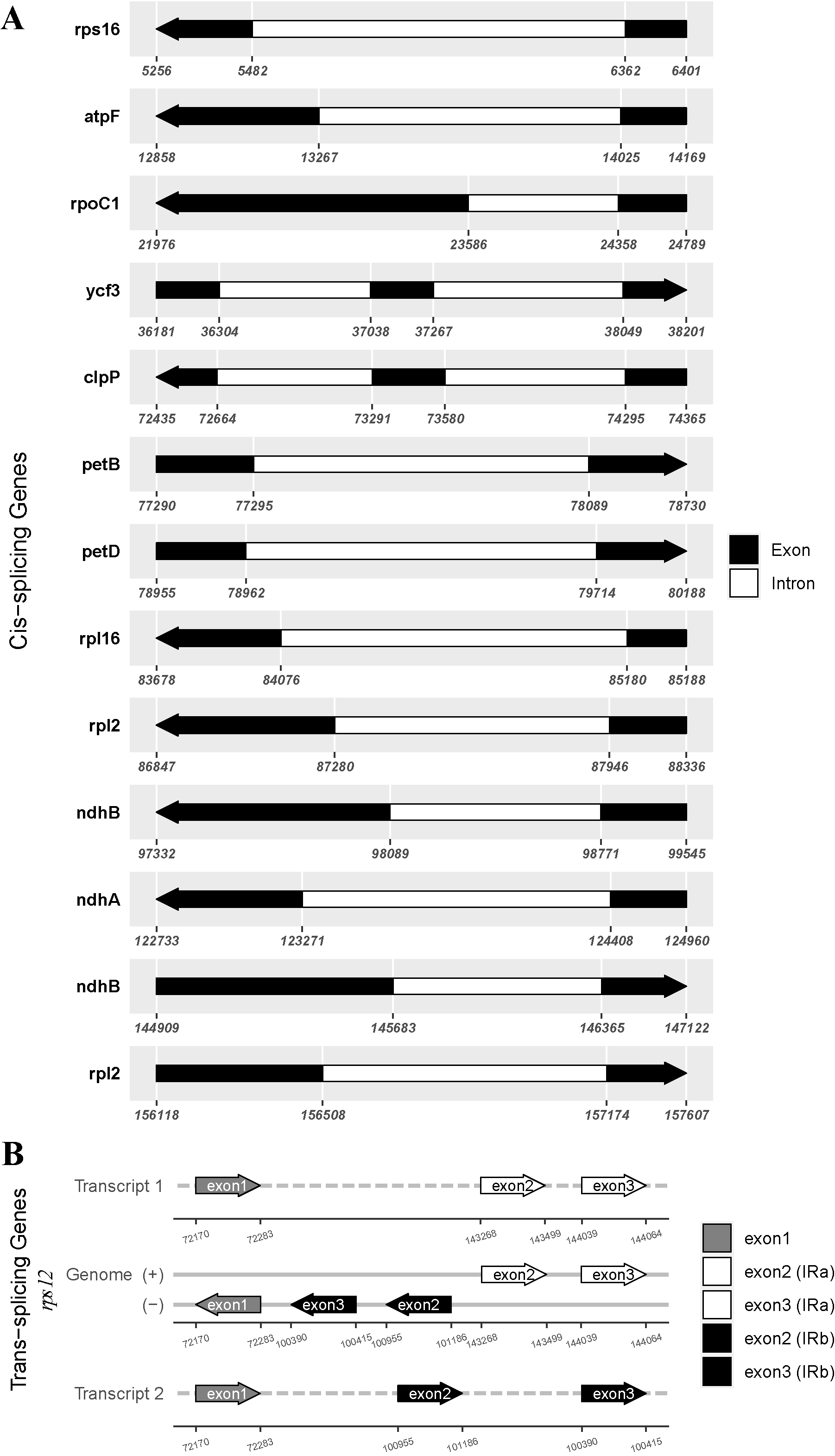


Figure S2. (A) Cis-splicing and (B) trans-splicing gene maps of *Mangifera indica* ‘Guiqi’.


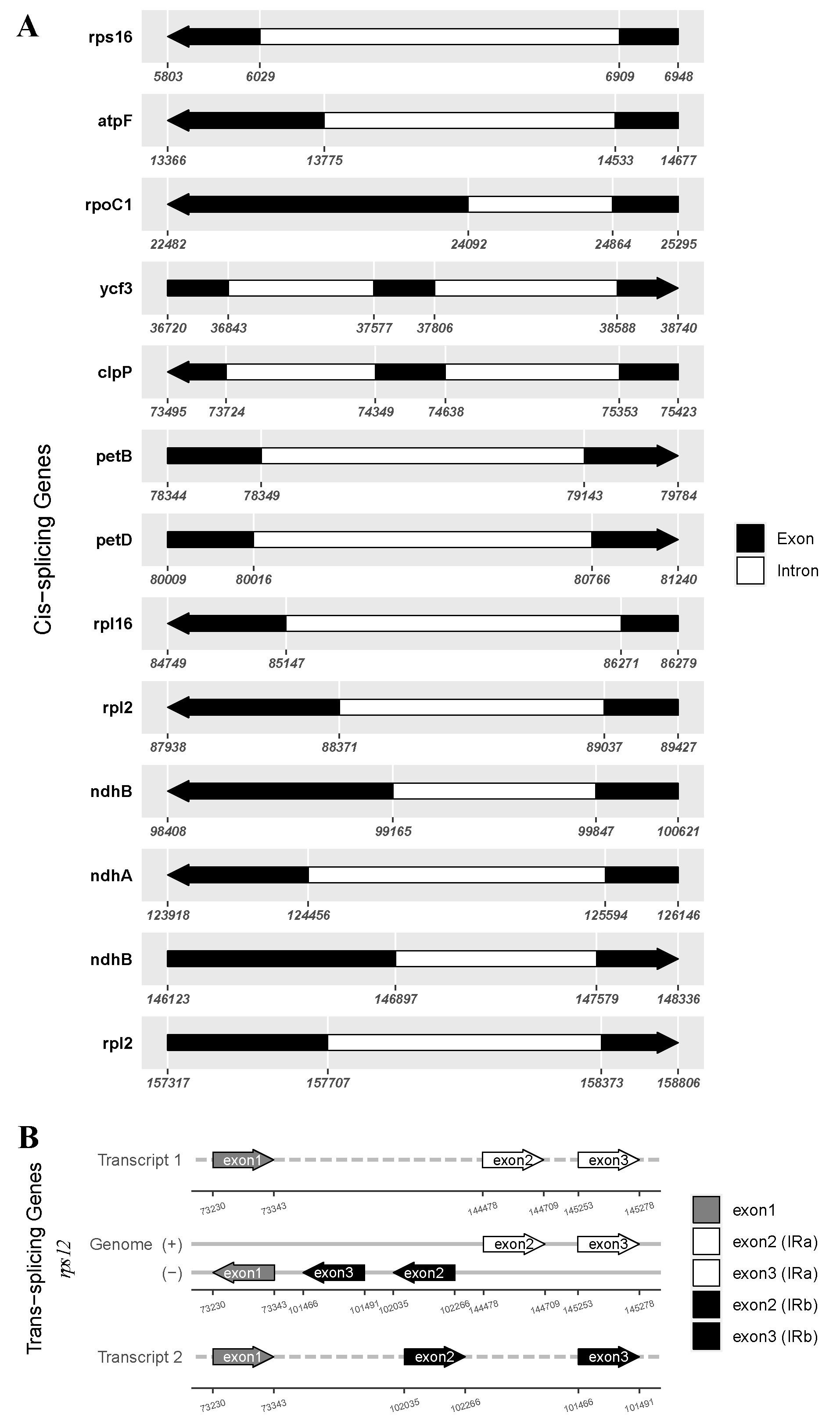


Figure S3. (A) Cis-splicing and (B) trans-splicing gene maps of *Mangifera quadrifida*.


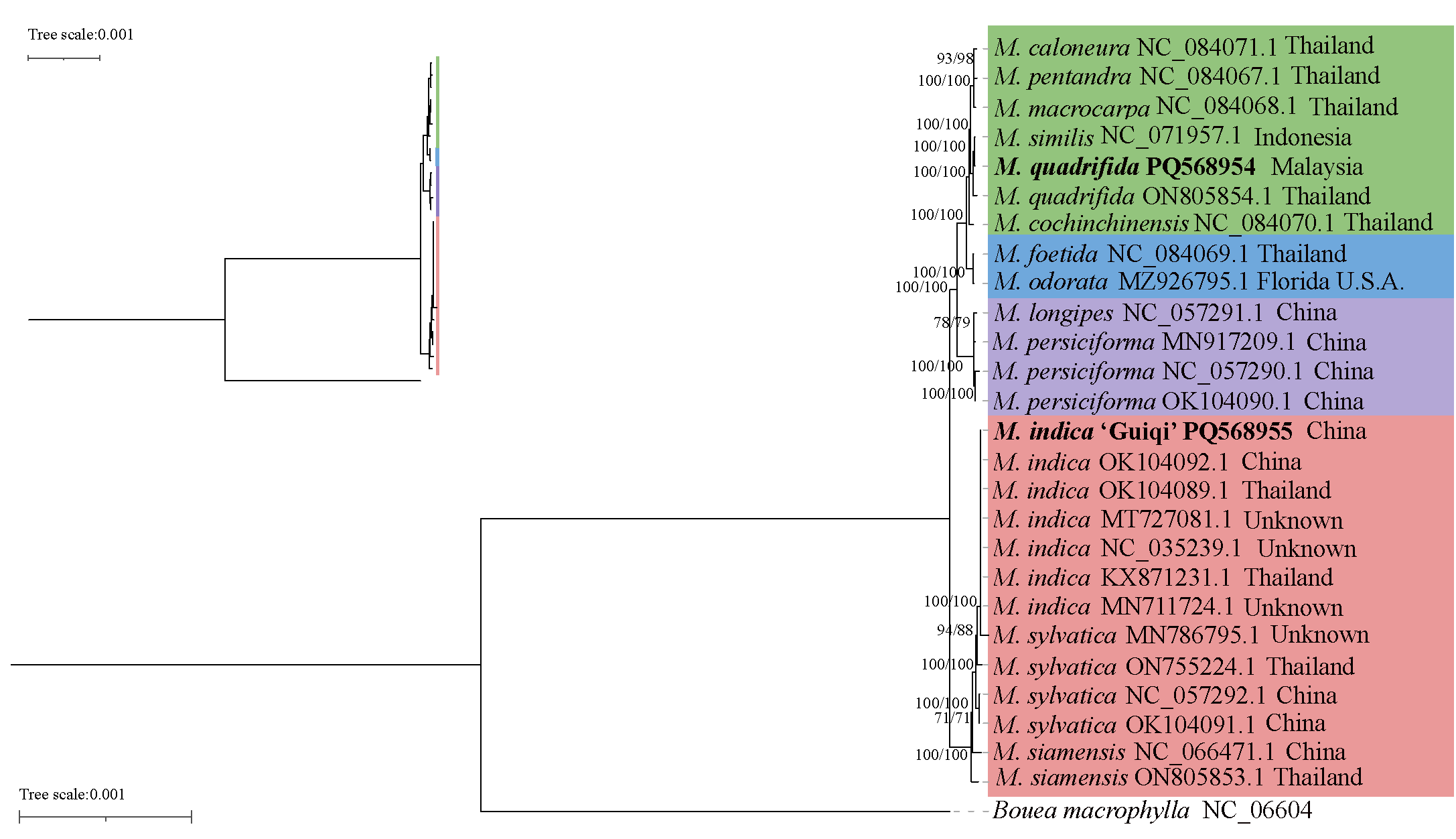


Figure S4. Maximum-likelihood phylogenetic tree of 26 *Mangifera* accessions and *Bouea macrophylla* as the outgroup based on the complete chloroplast genome sequences. Branch supports were calculated using the Shimodaira-Hasegawa-like approximate likelihood ratio test (SH-aLRT, left) and the UltraFast bootstraps (UFboot, right). The GenBank accession numbers and country of origin were listed behind the species name, while other information is available in Table S1.

Table S1. The accession numbers, collections/locations, the corresponding references, and the characteristics of chloroplast genomes used in this study.

| **Taxa** | **Accession numbers** | **Collections/Locations** | **References** | **Length (bp)** | **LSC (bp)** | **IRs (bp)** | **SSC (bp)** | **GC content (%)** | **Gene** | **CDS** | **rRNA** | **tRNA** |
| --- | --- | --- | --- | --- | --- | --- | --- | --- | --- | --- | --- | --- |
| *Mangifera indica ‘*Guiqi’ | PQ568955 | Nanning, Guangxi, China | **This study** | 157780 | 86673 | 26379 | 18349 | 37.9 | 133 | 87 | 8 | 37 |
| *M. quadrifida* | PQ568954 | TCM garden, INTI International University, Nilai, Negeri Sembilan, Malaysia (Seedling seed from Royal Belum Forest Reserve, Perak, Malaysia) | **This study** | 158979 | 87764 | 26396 | 18423 | 37.8 | 133 | 87 | 8 | 37 |
| *M. caloneura* | NC_084071 | Pak Thong Chai, Nakhon Ratchasima,Thailand | Xin Y, Yu WB, Eiadthong W, Cao Z, Li Q, Yang Z, Zhao W, Xin P. 2023. Comparative Analyses of 18 Complete Chloroplast Genomes from Eleven Mangifera Species (Anacardiaceae): Sequence Characteristics and Phylogenomics. Horticulturae. 9(1):86. https://doi.org/10.3390/horticulturae9010086 | 158931 | 87727 | 26387 | 18430 | 37.8 | 129 | 84 | 8 | 37 |
| *M. cochinchinensis* | NC_084070 | Soi Dao, Chantaburi,Thailand | Xin Y, Yu WB, Eiadthong W, Cao Z, Li Q, Yang Z, Zhao W, Xin P. 2023. Comparative Analyses of 18 Complete Chloroplast Genomes from Eleven Mangifera Species (Anacardiaceae): Sequence Characteristics and Phylogenomics. Horticulturae. 9(1):86. https://doi.org/10.3390/horticulturae9010086 | 158932 | 87735 | 26390 | 18417 | 37.8 | 129 | 84 | 8 | 37 |
| *M. foetida* | NC_084069 | Khao Chong, Nayong, Trang,Thailand | Xin Y, Yu WB, Eiadthong W, Cao Z, Li Q, Yang Z, Zhao W, Xin P. 2023. Comparative Analyses of 18 Complete Chloroplast Genomes from Eleven Mangifera Species (Anacardiaceae): Sequence Characteristics and Phylogenomics. Horticulturae. 9(1):86. https://doi.org/10.3390/horticulturae9010086 | 158887 | 87707 | 26377 | 18426 | 37.8 | 129 | 84 | 8 | 37 |
| *M. indica* | OK104089 | Chiengmai, Thailand | Tang YJ, Luo SX, Zhang Y, Zhao Y, Li RW, Guo LM, Huang GD, Gao AP, Huang JF. 2022. Comparative analysis of the structure of complete chloroplast genomes in genus Mangifera and accuracy verification about phylogenetic analysis based on gene ycf2 in genus level. bioRxiv. 2022:04.487216. https://doi.org/10.1101/2022.04.05.487216 | 157782 | 86674 | 26379 | 18350 | 37.9 | 133 | 88 | 8 | 37 |
| *M. indica* | OK104092 | Baise, Guangxi, Ghina | Tang YJ, Luo SX, Zhang Y, Zhao Y, Li RW, Guo LM, Huang GD, Gao AP, Huang JF. 2022. Comparative analysis of the structure of complete chloroplast genomes in genus Mangifera and accuracy verification about phylogenetic analysis based on gene ycf2 in genus level. bioRxiv. 2022:04.487216. https://doi.org/10.1101/2022.04.05.487216 | 157780 | 86673 | 26379 | 18349 | 37.9 | 133 | 88 | 8 | 37 |
| *M. indica* | KX871231 | Korea University greenhouse (Originated from Thailand) | Jo S, Kim HW, Kim YK, Sohn JY, Cheon SH, Kim KJ. 2017. The complete plastome sequences of Mangifera indica L. (Anacardiaceae). Mitochondrial DNA Part B. 2(2):698–700. https://doi.org/10.1080/23802359.2017.1390407 | 157780 | 86673 | 26379 | 18349 | 37.9 | 132 | 87 | 8 | 37 |
| *M. indica* | MN711724 | Unknown | Liang, C., Xu, J. and Chen, S. 2020. (unpublished) | 157775 | 86664 | 26381 | 18349 | 37.9 | 128 | 83 | 8 | 37 |
| *M. indica* | MT727081 | Unknown | Jiang, B. 2021. (unpublished) | 157779 | 86672 | 26379 | 18349 | 37.9 | 132 | 87 | 8 | 37 |
| *M. indica* | NC_035239 | Unknown | Rabah SO, Lee C, Hajrah NH, Makki RM, Alharby HF, Alhebshi AM, Sabir JSM, Jansen RK, Ruhlman TA. 2017. Plastome Sequencing of Ten Nonmodel Crop Species Uncovers a Large Insertion of Mitochondrial DNA in Cashew. Plant Genome. 10(3):10-14. https://doi.org/10.3835/plantgenome2017.03.0020 | 157780 | 86673 | 26379 | 18349 | 37.9 | 128 | 83 | 8 | 37 |
| *M. longipes* | NC_057291 | Xishuangbanna Tropical Botanical Garden of Chinese Academy of Sciences, Yunnan, China | Niu YF, Gao CW, Liu J. 2021. Comparative analysis of the complete plastid genomes of Mangifera species and gene transfer between plastid and mitochondrial genomes. PeerJ 9:e10774 https://doi.org/10.7717/peerj.10774 | 157853 | 86726 | 26379 | 18349 | 37.9 | 130 | 86 | 8 | 36 |
| *M. macrocarpa* | NC_084068 | Khao Chong, Nayong, Trang, Thailand | Xin Y, Yu WB, Eiadthong W, Cao Z, Li Q, Yang Z, Zhao W, Xin P. 2023. Comparative Analyses of 18 Complete Chloroplast Genomes from Eleven Mangifera Species (Anacardiaceae): Sequence Characteristics and Phylogenomics. Horticulturae. 9(1):86. https://doi.org/10.3390/horticulturae9010086 | 158942 | 87732 | 26387 | 18436 | 37.8 | 129 | 84 | 8 | 37 |
| *M. odorata* | MZ926795 | Florida, U.S.A | Tang YJ, Luo SX, Zhang Y, Zhao Y, Li RW, Guo LM, Huang GD, Gao AP, Huang JF. 2022. Comparative analysis of the structure of complete chloroplast genomes in genus Mangifera and accuracy verification about phylogenetic analysis based on gene ycf2 in genus level. bioRxiv. 2022:04.487216. https://doi.org/10.1101/2022.04.05.487216 | 158889 | 87708 | 26377 | 18427 | 37.8 | 133 | 88 | 8 | 37 |
| *M. pentandra* | NC_084067 | Khao Chong, Nayong, Trang, Thailand | Xin Y, Yu WB, Eiadthong W, Cao Z, Li Q, Yang Z, Zhao W, Xin P. 2023. Comparative Analyses of 18 Complete Chloroplast Genomes from Eleven Mangifera Species (Anacardiaceae): Sequence Characteristics and Phylogenomics. Horticulturae. 9(1):86. https://doi.org/10.3390/horticulturae9010086 | 158918 | 87710 | 26388 | 18432 | 37.8 | 129 | 84 | 8 | 37 |
| *M. persiciforma* | MN917209 | Xishuangbanna Tropical Botanical Garden of Chinese Academy of Sciences, Yunnan, China | Niu YF, Gao CW, Liu J. 2021. Comparative analysis of the complete plastid genomes of Mangifera species and gene transfer between plastid and mitochondrial genomes. PeerJ 9:e10774 https://doi.org/10.7717/peerj.10774 | 157799 | 86724 | 26354 | 18367 | 37.9 | 129 | 84 | 8 | 37 |
| *M. persiciforma* | NC_057290 | Xishuangbanna Tropical Botanical Garden of Chinese Academy of Sciences, Yunnan, China | Niu YF, Gao CW, Liu J. 2021. Comparative analysis of the complete plastid genomes of Mangifera species and gene transfer between plastid and mitochondrial genomes. PeerJ 9:e10774 https://doi.org/10.7717/peerj.10774 | 157796 | 86718 | 26355 | 18368 | 37.9 | 128 | 83 | 8 | 37 |
| *M. persiciforma* | OK104090 | Baise, Guangxi, Ghina | Tang YJ, Luo SX, Zhang Y, Zhao Y, Li RW, Guo LM, Huang GD, Gao AP, Huang JF. 2022. Comparative analysis of the structure of complete chloroplast genomes in genus Mangifera and accuracy verification about phylogenetic analysis based on gene ycf2 in genus level. bioRxiv. 2022:04.487216. https://doi.org/10.1101/2022.04.05.487216 | 158838 | 87566 | 26368 | 18536 | 37.9 | 133 | 88 | 8 | 37 |
| *M. quadrifida* | ON805854 | Khao Chong, Nayong, Trang, Thailand | Xin Y, Yu WB, Eiadthong W, Cao Z, Li Q, Yang Z, Zhao W, Xin P. 2023. Comparative Analyses of 18 Complete Chloroplast Genomes from Eleven Mangifera Species (Anacardiaceae): Sequence Characteristics and Phylogenomics. Horticulturae. 9(1):86. https://doi.org/10.3390/horticulturae9010086 | 158940 | 87731 | 26392 | 18425 | 37.8 | 129 | 84 | 8 | 37 |
| *M. siamensis* | NC_066471 | Nanning, Guangxi, China | Yuskianti V, Priyadi A, Ria R, Matra DD, Sadikin R, Rahayu S. 2024. The complete chloroplast genome of Mangifera similis Blume (Anacardiaceae) from Bangka Island, Indonesia. Journal of Asia-Pacific Biodiversity. 17(2):351-356. https://doi.org/10.1016/j.japb.2023.12.012. | 157604 | 86507 | 26389 | 18319 | 37.9 | 133 | 88 | 8 | 37 |
| *M. siamensis* | ON805853 | Lansak, Uthaithani, Thailand | Xin Y, Yu WB, Eiadthong W, Cao Z, Li Q, Yang Z, Zhao W, Xin P. 2023. Comparative Analyses of 18 Complete Chloroplast Genomes from Eleven Mangifera Species (Anacardiaceae): Sequence Characteristics and Phylogenomics. Horticulturae. 9(1):86. https://doi.org/10.3390/horticulturae9010086 | 158025 | 86856 | 26391 | 18387 | 37.8 | 129 | 84 | 8 | 37 |
| *M. similis* | NC_071957 | Bogor Botanical Garden (Originated from Bangka Island, Indonesia) | Yuskianti V , Priyadi A , Cahyaningsih R ,et al.The complete chloroplast genome of Mangifera similis Blume (Anacardiaceae) from Bangka Island, Indonesia[J].Journal of Asia-Pacific Biodiversity, 2024, 17(2):351-356.DOI:10.1016/j.japb.2023.12.012. | 158977 | 87764 | 26395 | 18423 | 37.8 | 132 | 87 | 8 | 37 |
| *M. sylvatica* | MN786795 | Unknown | Yang, G. and Zhang, Y. (unpublished) | 158106 | 87008 | 26379 | 18340 | 37.9 | 129 | 84 | 8 | 37 |
| *M. sylvatica* | NC_057292 | Xishuangbanna Tropical Botanical Garden of Chinese Academy of Sciences, Yunnan, China | Niu YF, Gao CW, Liu J. 2021. Comparative analysis of the complete plastid genomes of Mangifera species and gene transfer between plastid and mitochondrial genomes. PeerJ 9:e10774 https://doi.org/10.7717/peerj.10774 | 157824 | 86719 | 26379 | 18347 | 37.9 | 131 | 87 | 8 | 36 |
| *M. sylvatica* | OK104091 | Nanning, Guangxi, China | Niu YF, Gao CW, Liu J. 2021. Comparative analysis of the complete plastid genomes of Mangifera species and gene transfer between plastid and mitochondrial genomes. PeerJ 9:e10774 https://doi.org/10.7717/peerj.10774 | 157739 | 86672 | 26360 | 18347 | 37.9 | 133 | 88 | 8 | 37 |
| *M. sylvatica* | ON755224 | Umphang, Tak, Thailand | Xin Y, Yu WB, Eiadthong W, Cao Z, Li Q, Yang Z, Zhao W, Xin P. 2023. Comparative Analyses of 18 Complete Chloroplast Genomes from Eleven Mangifera Species (Anacardiaceae): Sequence Characteristics and Phylogenomics. Horticulturae. 9(1):86. https://doi.org/10.3390/horticulturae9010086 | 157368 | 86228 | 26396 | 18348 | 37.9 | 129 | 84 | 8 | 37 |
| *Bouea macrophylla* | NC_066047 | Unknown | Huang YH, Chen DJ and Wang HF. (unpublished) | 158826 | 87775 | 25993 | 19065 | 37.7 | 133 | 88 | 8 | 37 |
